# Development of mouse models of angiosarcoma driven by p53

**DOI:** 10.1242/dmm.038612

**Published:** 2019-07-09

**Authors:** Donald M. Salter, Meredyth Griffin, Morwenna Muir, Katy Teo, Jayne Culley, James R. Smith, Laura Gomez-Cuadrado, Kylie Matchett, Andrew H. Sims, Larry Hayward, Neil C. Henderson, Valerie G. Brunton

**Affiliations:** 1 Centre for Genomic & Experimental Medicine, Institute of Genetics & Molecular Medicine, University of Edinburgh, Crewe Road South, Edinburgh EH4 2XR, UK; 2 Edinburgh Cancer Research UK Centre, Institute of Genetics & Molecular Medicine, University of Edinburgh, Crewe Road South, Edinburgh EH4 2XR, UK; 3 Centre for Inflammation Research, The Queen's Medical Research Institute, University of Edinburgh, Little France Crescent, Edinburgh EH16 4TJ, UK

**Keywords:** Angiosarcoma, *Trp53*, Genetically engineered mouse model, Lymphomas, Tumour

## Abstract

Angiosarcomas are a rare group of tumours which have poor prognosis and limited treatment options. The development of new therapies has been hampered by a lack of good preclinical models. Here, we describe the development of an autochthonous mouse model of angiosarcoma driven by loss of p53 in VE-cadherin-expressing endothelial cells. Using *Cdh5-Cre* to drive recombination in adult endothelial cells, mice developed angiosarcomas with 100% penetrance upon homozygous deletion of *Trp53* with a median lifespan of 325 days. In contrast, expression of the R172H mutant p53 resulted in formation of thymic lymphomas with a more rapid onset (median lifespan 151 days). We also used *Pdgfrb-Cre*-expressing mice, allowing us to target predominantly pericytes, as these have been reported as the cell of origin for a number of soft tissue sarcomas. *Pdgfrb-Cre* also results in low levels of recombination in venous blood endothelial cells in multiple tissues during development. Upon deletion of *Trp53* in *Pdgfrb-Cre*-expressing mice (*Pdgfrb-Cre,* *Trp53^fl/fl^* mice), 65% developed lymphomas and 21% developed pleomorphic undifferentiated soft tissue sarcomas. None developed angiosarcomas. In contrast, 75% of *Pdgfrb-Cre,* *Trp53^R172H/R172H^* mice developed angiosarcomas, with 60% of these mice also developing lymphomas. The median lifespan of the *Pdgfrb-Cre,* *Trp53^R172H/R172H^* mice was 151 days. Re-implantation of angiosarcoma tumour fragments from *Cdh5-Cre, Trp53^fl/fl^* mice provided a more consistent and rapid model of angiosarcoma than the two spontaneous models. The ability to passage tumour fragments through the mouse provides a novel model which is amenable to preclinical studies and will help the development of potential new therapies for angiosarcoma.

## Abstract (precis)

**Editor's choice:** Deletion of p53, rather than mutation, in endothelial cells leads to reliable angiosarcoma generation, which, along with establishment of a transplantation model, provides a novel approach for testing potential new therapeutics.

## INTRODUCTION

Angiosarcomas are rare but aggressive endothelial cell tumours. Most arise spontaneously, but they also develop following ionizing radiation and chronic lymphoedema. They have a high risk of local recurrence and metastasis, with limited treatment options such that the overall five year survival is ∼35% (Young et al., 2010). They typically express endothelial markers such as CD31 (also known as PECAM1) and vascular endothelial growth factor (VEGF) and, for this reason, there is interest in the use of anti-angiogenic therapies for their treatment. Data from a number of clinical trials show promising activity of the VEGFA monoclonal antibody bevacizumab and broad-spectrum small molecule tyrosine kinase inhibitors that target VEGF receptors (Young et al., 2010). However, the underlying pathways driving the pathogenesis of angiosarcoma are not well defined. To address this, and the urgent need for effective therapies, we set out to develop an autochthonous mouse model of angiosarcoma that could aid preclinical drug development efforts.

Mutations in *TP53* (which encodes p53) have been reported in human angiosarcomas, with incidences of between 4% and 52% reported in different studies (Behjati et al., 2014; Hung et al., 2013; Italiano et al., 2012; Murali et al., 2015; Naka et al., 1997; Weihrauch et al., 2002; Zietz et al., 1998). In addition, mice with germline deletion of *Trp53* which are predisposed to the development of lymphoma develop angiosarcomas in significant numbers (Donehower et al., 1992; Jacks et al., 1994). The predominance and rapid development of lymphoma in these models precludes their usefulness as models of angiosarcoma. Attempts have therefore been made to overcome this by use of tissue-specific Cre-Lox recombination of conditional alleles and alymphocytic mice (Farhang Ghahremani et al., 2014; Landuzzi et al., 2014).

There is increasing evidence that mesenchymal stem cells (MSCs) may be the cell of origin of a number of different sarcoma subtypes (Charytonowicz et al., 2009; Matushansky et al., 2007; Riggi et al., 2005; Rubio et al., 2010; Sato et al., 2016; Tirode et al., 2007). Coupled with the evidence that pericytes have been proposed as the identity of MSCs in normal tissues (Caplan, 2008; Crisan et al., 2008) and some sarcomas share features of pericytes, such as expression of NG2 (CSPG4) and CD146 (MCAM) (Benassi et al., 2009; Wei et al., 2015), we set out to address whether *Pdgfrb-Cre* and *Cdh5-CreER^T2^* mice could be used to generate a model of angiosarcoma. *Pdgfrb-Cre* mice express Cre recombinase under the control of a fragment of the gene that encodes platelet-derived growth factor receptor-β (PDGFR-β). This drives Cre-mediated recombination in pericytes in a number of tissues (Foo et al., 2006; Henderson et al., 2013) and low levels of recombination in venous blood endothelial cells in multiple tissues during development (Stanczuk et al., 2015; Ulvmar et al., 2016). This allows us to target loss of p53 function to the pericyte lineage while also potentially targeting endothelial cell lineages. *Cdh5-CreER^T2^* mice express Cre recombinase under the inducible control of the vascular endothelial cadherin (VE-cadherin; encoded by *Cdh5*) promoter in adult mice to drive expression in endothelial cells (Wang et al., 2010).

It is widely accepted that mutant forms of p53 can exert dominant negative or gain-of-function effects that contribute to tumour development beyond that seen following loss of the wild-type p53 protein alone (Blagosklonny, 2000; Sigal and Rotter, 2000). Individuals with Li-Fraumeni syndrome carry inherited mutations in *TP53* and are predisposed to tumour development, including sarcomas. In mouse models of Li-Fraumeni syndrome, expression of *Trp53^R172H^*, which corresponds to the *TP53^R175H^* hotspot mutation in human tumours, in mice leads to the development of predominantly lymphomas, but a small percentage of these mice also develop angiosarcomas (Lang et al., 2004; Olive et al., 2004). We therefore generated mice in which *Trp53^R172H^* was expressed under the control of *Pdgfrb-Cre* and *Cdh5-CreER^T2^*, in addition to those carrying a floxed *Trp53* allele.

## RESULTS

### Tumour development in *Pdgfrb-Cre,* *Trp53^R172H/R172H^* and *Pdgfrb-Cre,* *Trp53^fl/fl^* mice

**Fig. 1.**
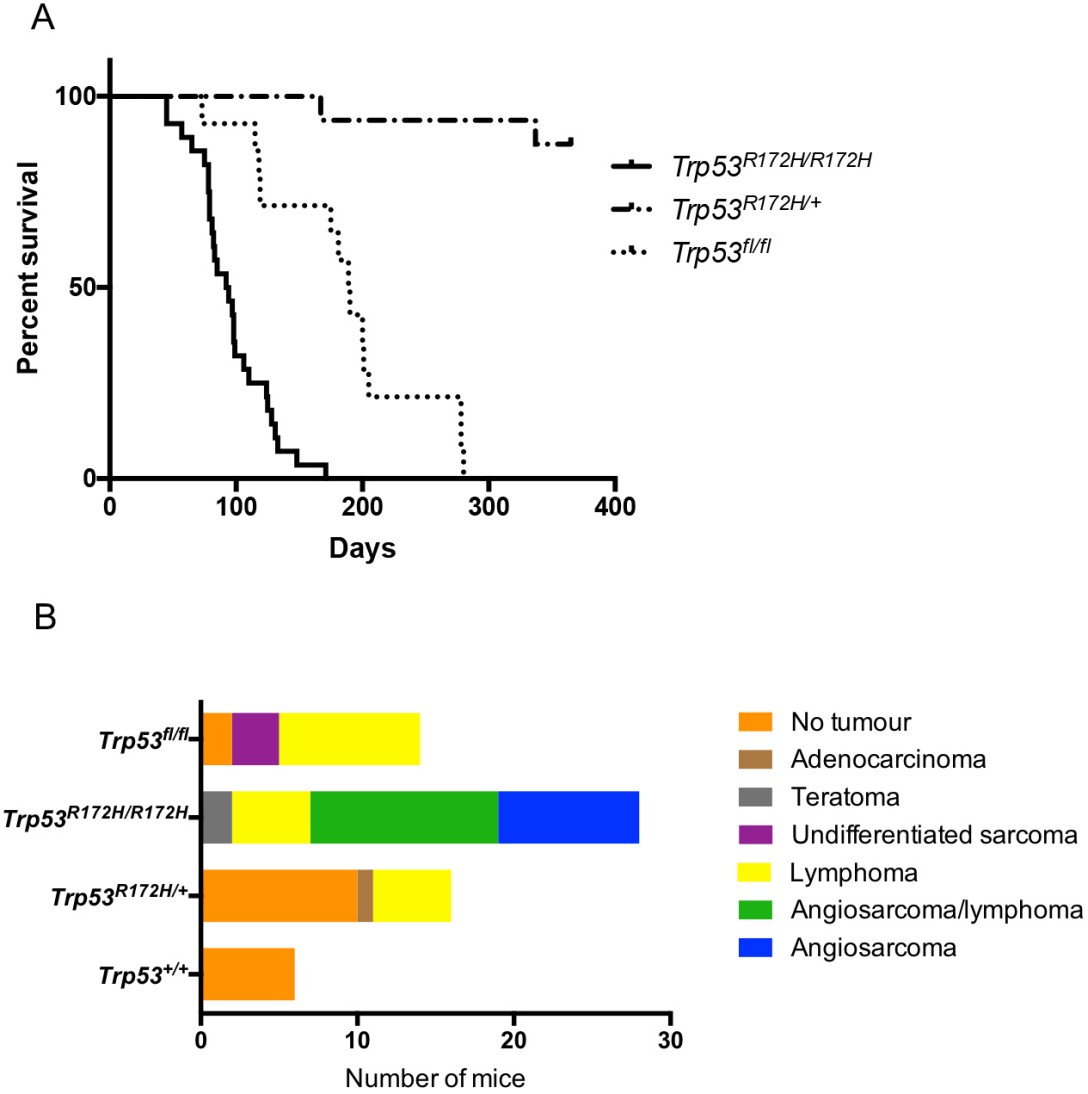
**Tumour development in *Pdgfrb-Cre* mice.** (A) Kaplan–Meier curves showing significant difference in survival between *Trp53^R172H/R172H^* (*n*=28), *Trp53^R172H/+^* (*n*=16) and Trp53^fl/fl^ (*n*=14) mice (log-rank *P*<0.0001). (B) Tumour incidence and type in the different mouse cohorts.

Experimental cohorts consisted of mice expressing either one (*Pdgfrb-Cre, Trp53^R172H/+^*) (*n*=16) or two (*Pdgfrb-Cre, Trp53^R172H/R172H^*) (*n*=28) mutant *Trp53^R172H^* alleles, or loss of both *Trp53* alleles (*Pdgfrb-Cre, Trp53^fl/fl^*) (*n*=14). The median lifespan of the *Pdgfrb-Cre, Trp53^R172H/R172H^* mice was 93 days compared to >365 days for the *Pdgfrb-Cre, Trp53^R172H/+^* mice and 189.5 days for the *Pdgfrb-Cre, Trp53^fl/fl^* mice (Fig. 1A). The deaths of all *Pdgfrb-Cre, Trp53^R172H/R172H^* mice were due to tumour formation, in contrast to the *Pdgfrb-Cre, Trp53^R172H/+^* cohort in which only 2/16 mice were culled owing to tumour formation. In the *Pdgfrb-Cre, Trp53^fl/fl^* cohort, 12/14 mice were culled owing to tumour formation. Mice that were asymptomatic at 1 year of age were culled.

### Autopsy findings and tumour histology

In the *Pdgfrb-Cre, Trp53^R172H/R172H^* cohort, 75% (*n*=21/28) of the mice developed angiosarcomas. Of these 21 mice, nine demonstrated only angiosarcomas, whereas in the other 12 mice lymphomas were also identified. The remaining *Pdgfrb-Cre, Trp53^R172H/R172H^* mice developed either lymphomas (*n*=5/28) or teratomas (*n*=2/28) (Fig. 1B). Thus, the predominant tumour type was angiosarcoma, with most mice developing multiple angiosarcomas in a number of different organs (Table 1) (Fig. 1B: median 3, range 1-6 tumours). No angiosarcomas were seen in the *Pdgfrb-Cre, Trp53^R172H/+^* mice. The two *Pdgfrb-Cre, Trp53^R172H/+^* mice culled owing to tumour formation had developed lymphomas. At autopsy, following culling of the asymptomatic *Pdgfrb-Cre, Trp53^R172H/+^* mice at 1 year, three were found to have developed lymphomas and one was found to have adenocarcinoma in the lung. The remainder showed no gross or histological abnormality (Fig. 1B). Within the *Pdgfrb-Cre, Trp53^fl/fl^* mice, upon histological examination it was found that nine developed lymphomas and three developed undifferentiated sarcomas; the remaining two had no detectable tumour upon sacrifice (Fig. 1B).

**Table 1.**
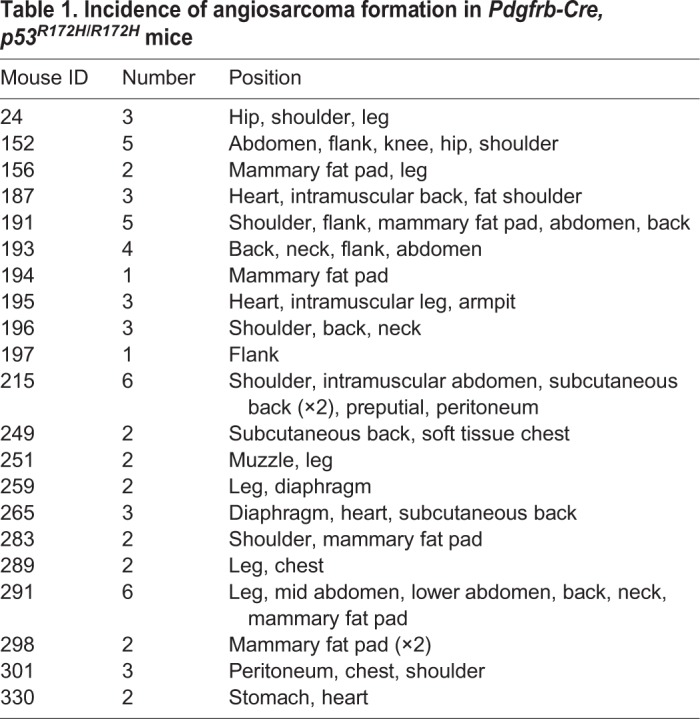
**Incidence of angiosarcoma formation in *Pdgfrb-Cre, p53^R172H/R172H^* mice**

### Characterization of angiosarcomas in *Pdgfrb-Cre, Trp53^R172H/R172H^* mice

**Fig. 2.**
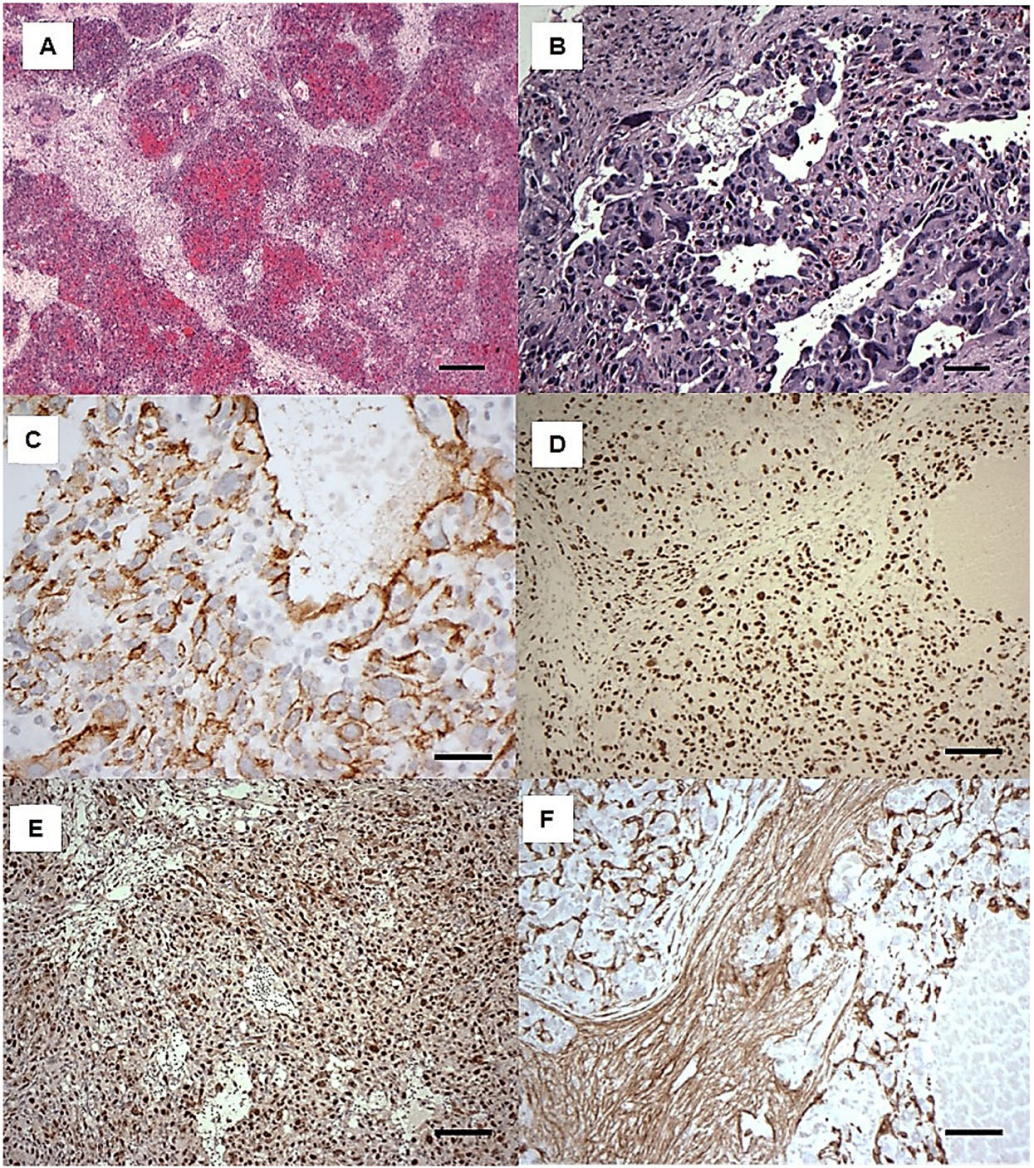
**Histology and immunohistochemistry of mouse angiosarcomas in *Pdgfrb-Cre, Trp53^R172H/R172H^* mice.** (A,B) H&E staining showing vascular lobulated tumour masses. These were composed of pleomorphic tumour cells showing variable vasoformative capability. (C-F) Immunohistochemical analysis with antibodies to CD31 (C), ERG (D), p53 (E) and PDGFR-β (F). Scale bars: 500 µm in A; 50 µm in B,C,F; 250 µm in D,E.

The morphological appearances of the angiosarcomas were similar within and between mice. The tumours consisted of lobules of pleomorphic cells showing varying degrees of vascular formation, which is typical of high-grade angiosarcomas (Fig. 2A,B). Immunohistochemistry supported the morphological assessment, with the tumour cells expressing CD31 and ERG (Fig. 2C,D). There was no expression of CD34 by the tumour cells (not shown). The tumours also showed strong expression of p53 (Fig. 2E) in keeping with the stabilization of mutant p53 that is often seen in human tumours expressing mutant p53. PDGFR-β was expressed by stromal cells within the tumour masses, but not reliably by the angiosarcomatous cells (Fig. 2F).

### Development of undifferentiated sarcomas in *Pdgfrb-Cre, Trp53^fl/fl^* mice

Three of the 14 (21%) *Pdgfrb-Cre, Trp53^fl/fl^* mice developed tumours with the morphological features of high-grade spindle cell and pleomorphic undifferentiated soft tissue sarcoma (Fig. S1A). Immunohistochemistry showed no expression of p53, confirming the homozygous deletion of p53 in the *Pdgfrb-Cre, Trp53^fl/fl^* mice (Fig. S1B), and strong expression of PDGFR-β by the tumour cells (Fig. S1C). Less than 10% of the cells expressed SMA (Acta2) in two of the cases, whereas the other was completely negative for SMA. None of the tumour cells expressed CD31 (Fig. S1D) or ERG (not shown).

### *Pdgfrb*-*Cre*-mediated recombination does not occur in adult CD31 endothelial cells

**Fig. 3.**
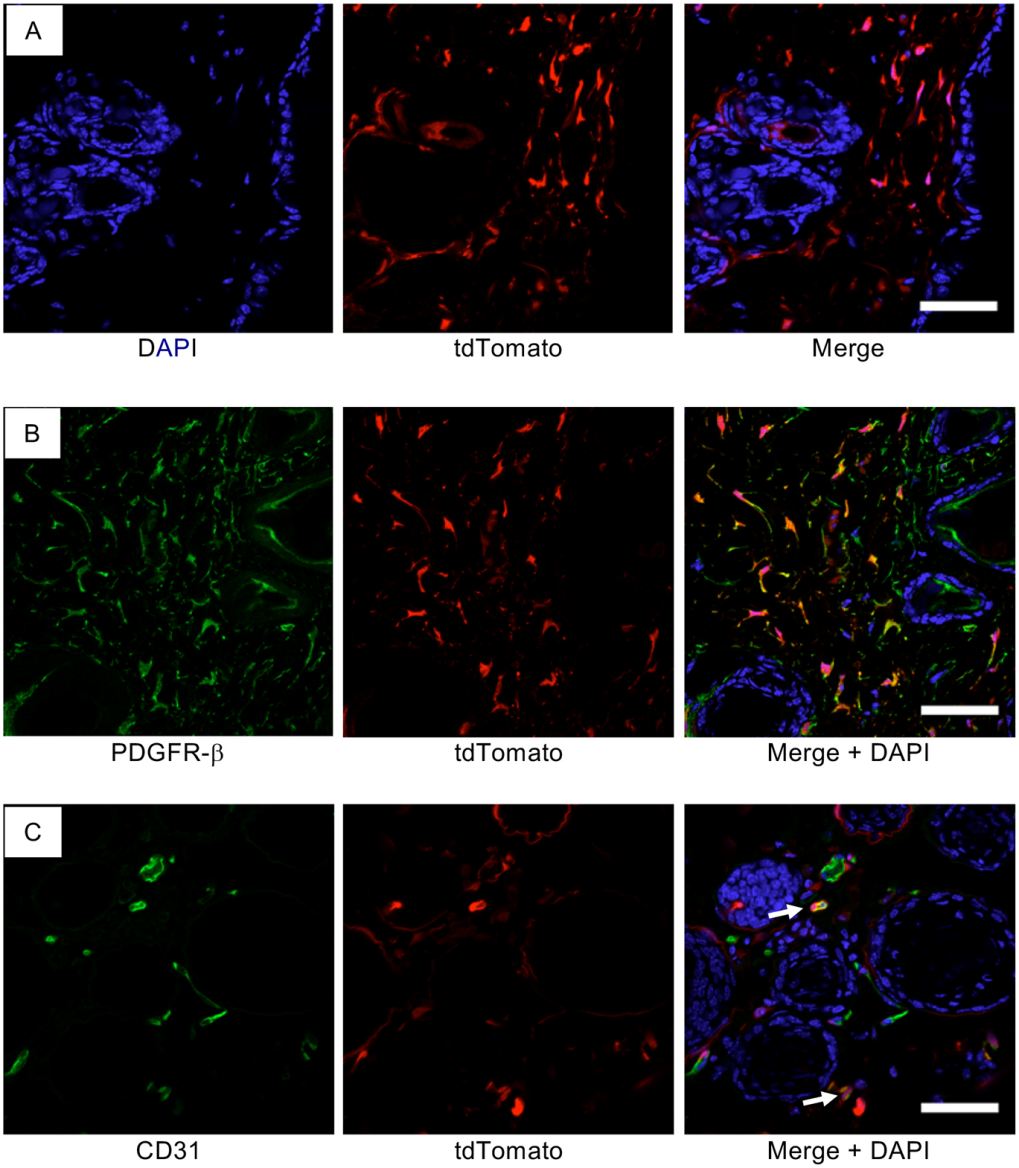
**PDGFR**-**β does not co-localize with CD31 in mouse skin.** (A-C) Confocal micrographs of fixed frozen dorsal skin from the *Pdgfrb-Cre* mouse crossed with a tdTomato floxed reporter (*Ai14*;*Pdgfrb-Cre*) showing tdTomato fluorescence (red; A-C), PDGFR-β antibody staining (green; B) and CD31 antibody staining (green; C). DAPI (blue) indicates nuclei. Arrows demonstrate CD31-labelled endothelial cells surrounded by a tdTomato-positive perivascular cell. Scale bars: 50 µm.

As the angiosarcomas that developed expressed CD31 but did not express PDGFR-β, we asked whether there was any Cre-mediated recombination in CD31-positive endothelial cells in adult mice. Using Ai14 reporter mice (single-fluorescent reporter mice that express tdTomato after Cre-mediated recombination) (Madisen et al., 2010), we found that *Pdgfrb-Cre* induced highly efficient recombination in mouse skin (Fig. 3A), a tissue in which a number of angiosarcomas arose in the *Pdgfrb-Cre, Trp53^R172H/R172H^* mice. To evaluate the specificity of recombination, we stained skin from Ai14;*Pdgfrb*-*Cre* mice for PDGFR-β and confirmed appropriate reporter expression using *Pdgfrb*-*Cre* (Fig. 3B). Staining of Ai14;*Pdgfrb*-*Cre* mice skin for CD31 expression showed that recombination did not target adult endothelial cells and, in some cases, PDGFR-β-expressing cells were seen surrounding small CD31-positive endothelial cells (Fig. 3C). This suggests that the angiosarcomas have arisen either from endothelial cell lineages that transiently express PDGFR-β during development (Stanczuk et al., 2015; Ulvmar et al., 2016) or from other PDGFR-β-expressing pericyte lineages. Interestingly the undifferentiated sarcomas that developed in the *Pdgfrb-Cre, Trp53^fl/fl^* mice had retained expression of PDGFR-β, suggesting a different cell of origin.

### Tumour development in *Cdh5-CreER^T2^, Trp53^R172H/R172H^* and *Cdh5-CreER^T2^, Trp53^fl/fl^* mice

**Fig. 4.**
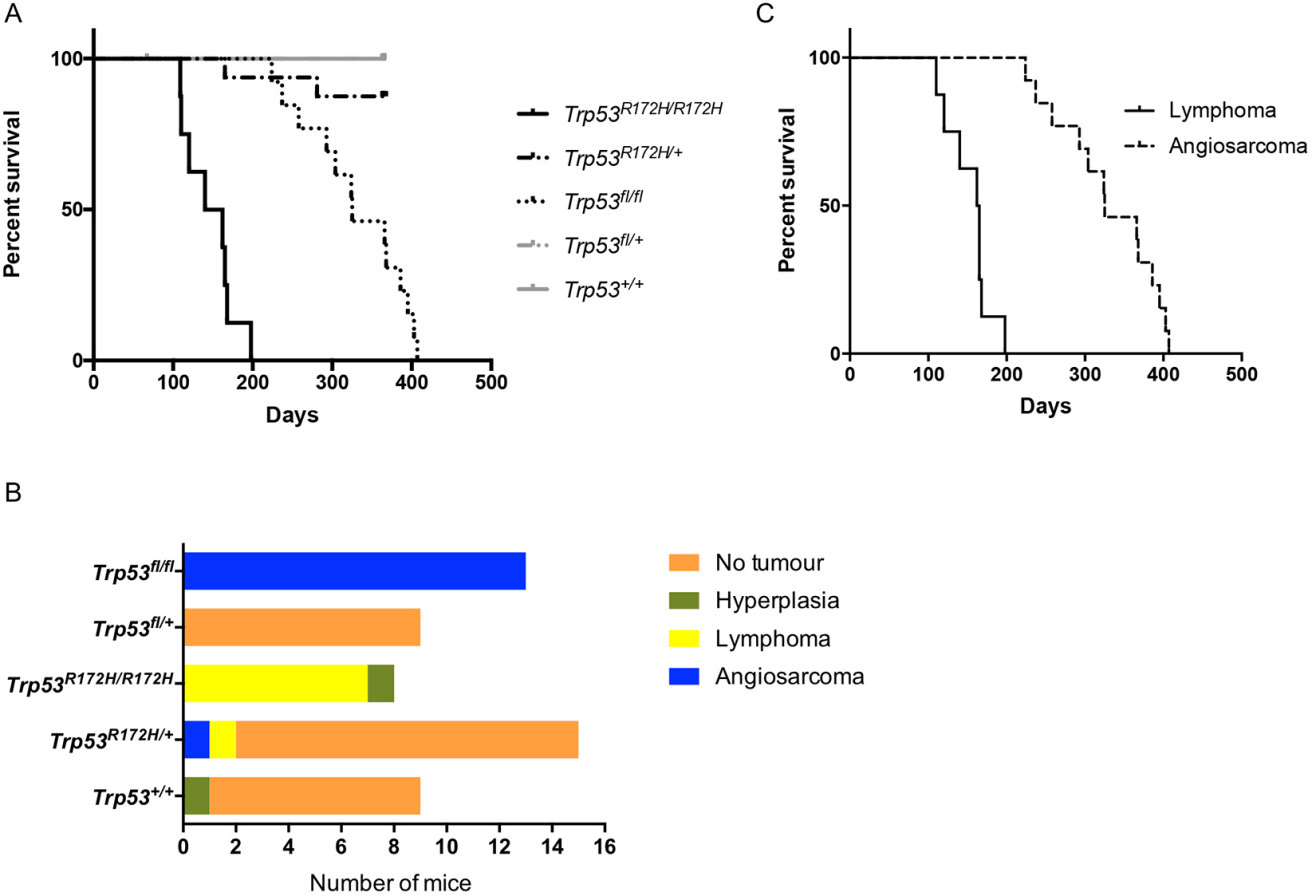
**Tumour development in *Cdh5-CreER^T2^* mice.** (A) Kaplan–Meier curves showing significant difference in survival between different mouse cohorts (log-rank *P*<0.0001). (B) Tumour incidence and type in the different mouse cohorts. (C) Kaplan–Meier curves showing significant difference in survival between mice developing angiosarcomas and lymphomas (log-rank *P*<0.0001).

To determine whether we could promote more efficient generation of angiosarcomas, we directly induced expression of mutant p53 or loss of p53 in adult endothelial cells using *Cdh5-CreER^T2^* mice, in which Cre recombinase is driven by *Cdh5*. Using Ai14;*Cdh5*-*CreER^T2^* reporter mice we found that *Cdh5-Cre* induced recombination in CD31-positive endothelial cells (Fig. S2). Experimental cohorts consisted of mice expressing either one (*Cdh5-CreER^T2^, Trp53^R172H/+^*) (*n*=15) or two (*Cdh5-CreER^T2^, Trp53^R172H/R172H^*) (*n*=8) mutant *Trp53^R172H^* alleles, or loss of one (*Cdh5-CreER^T2^, Trp53^fl/+^*) (*n*=9) or both (*Cdh5-CreER^T2^, Trp53^fl/fl^*) (*n*=13) *Trp53* alleles. A control cohort of *Cdh5-CreER^T2^* mice were also treated with tamoxifen (*n*=9). The median lifespan of *Cdh5-CreER^T2^, Trp53^R172H/R172H^* mice was 151 days (range 109-198) (Fig. 4A). In the *Cdh5-CreER^T2^, Trp53^R172H/+^* cohort, 2/16 mice developed tumours; all other mice were asymptomatic and sacrificed at 1 year (Fig. 4A,B). The median lifespan of *Cdh5-CreER^T2^, Trp53^fl/fl^* mice was 325 days (range 224-407) (Fig. 4A). All were culled owing to tumour formation. All *Cdh5-CreER^T2^, Trp53^fl/+^* and *Cdh5-CreER^T2^* mice were asymptomatic at 12 months of age and culled with no evidence of tumour formation upon autopsy.

### Autopsy findings and tumour histology

In the *Cdh5-CreER^T2^, Trp53^R172H/R172H^* cohort, 7/8 mice developed thymic lymphomas, with evidence of thymic hyperplasia in the remaining mouse, but none developed angiosarcomas (Fig. 4B). Two of the mice that developed lymphomas also developed additional tumours: one an undifferentiated sarcoma and the other a hepatocellular carcinoma. In addition, one of the *Cdh5-CreER^T2^* mice showed evidence of thymic hyperplasia, with all the others showing no evidence of tumour formation upon autopsy (Fig. 4B). In the *Cdh5-CreER^T2^, Trp53^R172H/+^* mice, two developed tumours: one a thymic lymphoma and the other an angiosarcoma (Fig. 4B). At autopsy, following culling of the asymptomatic *Cdh5-CreER^T2^, Trp53^R172H/+^* mice at 1 year, the remainder showed no gross or histological abnormality. Thus, the predominant tumour type driven by *Trp53^R172H^* in the *Cdh5-CreER^T2^* mice was lymphoma, in contrast to the angiosarcomas that developed in the *Pdgfrb-Cre, Trp53^R172H/R172H^* mice. Within the *Cdh5-CreER^T2^, Trp53^fl/fl^* cohort all mice developed angiosarcomas (13/13), many with multiple tumours that developed in a range of anatomical locations (Table 2) (Fig. 4B). The *Cdh5-CreER^T2^, Trp53^fl/+^* mice had no detectable tumours upon sacrifice. When we looked at the latency of the angiosarcomas and the lymphomas in all experimental mice we found that the lymphomas developed more rapidly than the angiosarcomas, with a median survival of 163 days (range 109-198) days and 325 days (range 224-407), respectively (Fig. 4C).

**Table 2.**
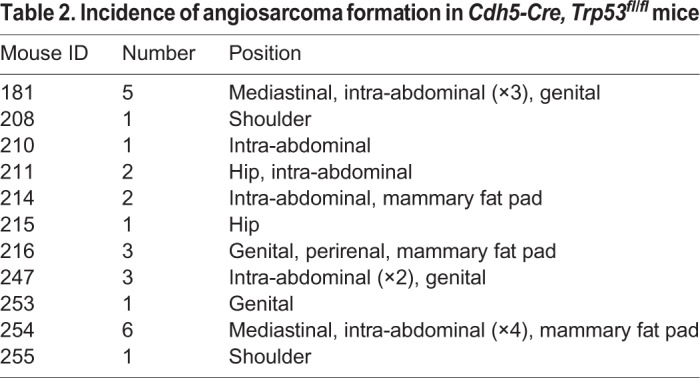
**Incidence of angiosarcoma formation in *Cdh5-Cre,Trp53^fl/fl^*** **mice**

### Characterization of angiosarcomas in *Cdh5-CreER^T2^, Trp53^fl/fl^* mice

**Fig. 5.**
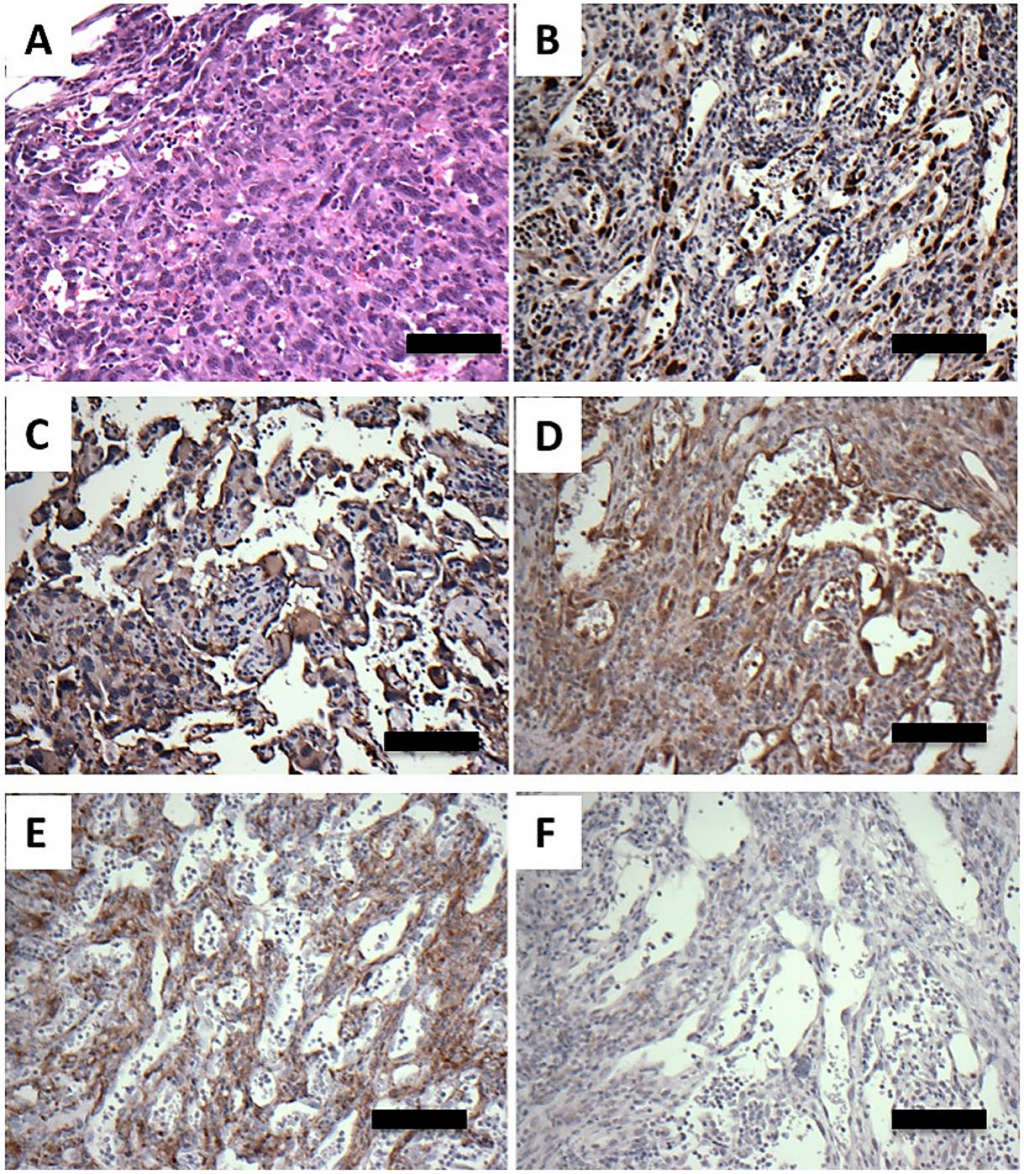
**Histology and immunohistochemistry of mouse angiosarcomas in *Cdh5-CreER^T2^, Trp53^fl/fl^* mice.** (A) H&E staining showing atypical cells lining vascular channels. (B-F) Immunohistochemical analysis using antibodies to ERG (B), CD31 (C), VE-cadherin (D), PDGFR-β (E) and p53 (F). Scale bars: 50 µm in A; 100 µm in B-F.

Many of the tumours were similar to those seen in the *Pdgfrb-Cre, Trp53^R172H/R172H^* mice, comprising lobules of pleomorphic cells showing varying degrees of vascular formation (Fig. 5A). However, many of the tumours showed extensive haemorrhage and necrosis. In some tumours, a cavernous/telangiectatic pattern was evident, with enlarged blood-filled spaces being lined by atypical endothelial cells. Immunohistochemistry supported the morphological assessment, with the tumour cells expressing ERG, CD31 and VE-cadherin, but not PDGFR-β (Fig. 5). Twelve of the 13 tumours that developed in the *Cdh5-CreER^T2^, Trp53^fl/fl^* mice did not express p53 (Fig. 5F). The reason for p53 expression in the remaining angiosarcoma is not known.

### Comparison of gene expression profiles of *Pdgfrb-Cre, Trp53^R172H/R172H^* and *Cdh5-CreER^T2^, Trp53^fl/fl^* tumours

**Fig. 6.**
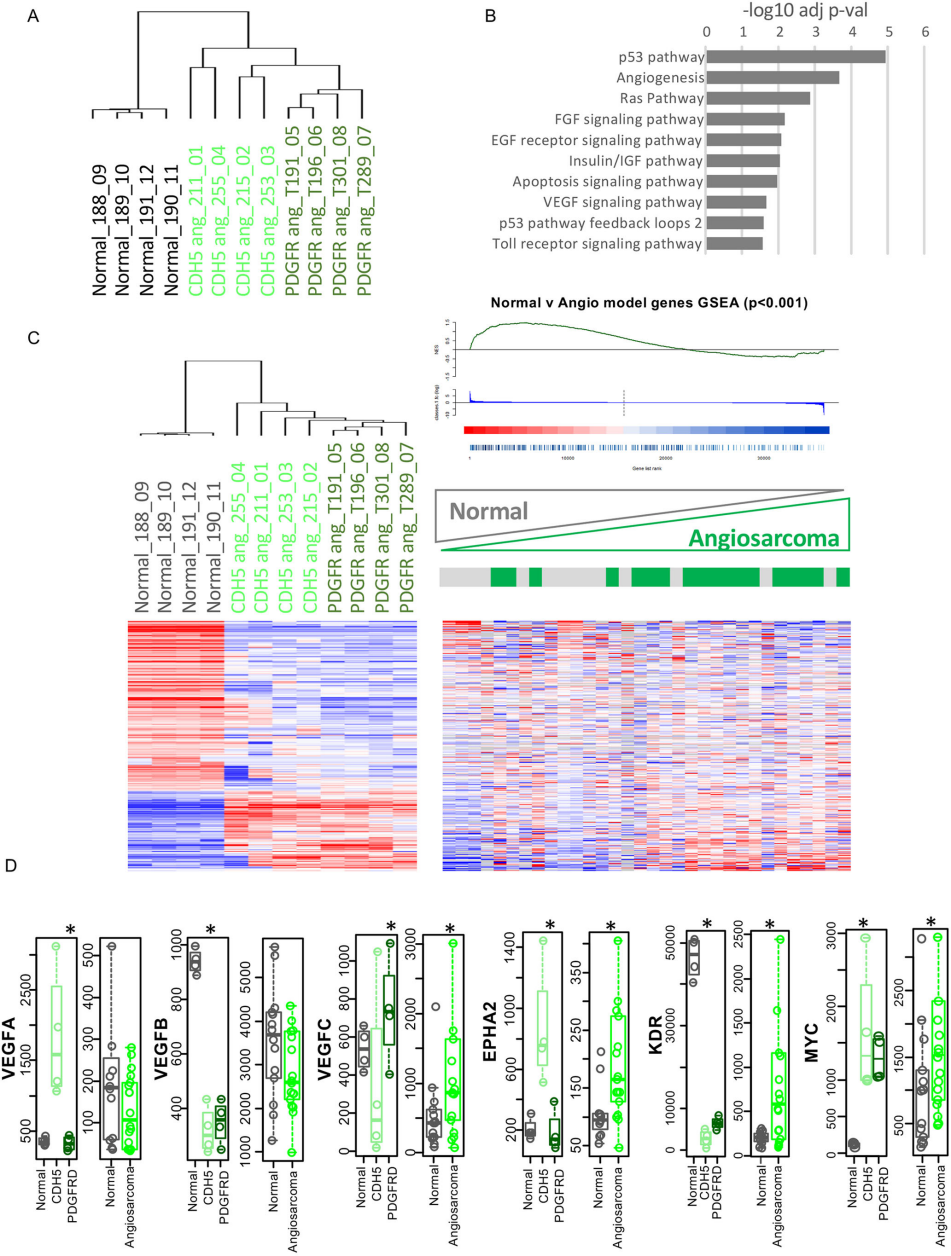
**Gene expression analysis demonstrates mouse angiosarcomas have common and distinct features, but largely resemble human angiosarcomas.** (A) Unsupervised hierarchical clustering of the NanoString PanCancer Pathways panel demonstrated some variation between the mouse angiosarcomas, but these are significantly more different than normal VE-cadherin (Cdh5)-derived endothelial cells. (B) Analysis of PANTHER signalling pathways that were significantly differentially expressed between the two mouse angiosarcomas. (C) The 299 significantly differently expressed genes between the mouse angiosarcomas and normal VE-cadherin-derived endothelial cells were significantly enriched in human angiosarcomas compared to normal human endothelial cells. (D) Genes associated with endothelial cell function were significantly increased in angiosarcomas in both the human and mouse datasets (VEGFC, EPHA2), whereas others were significantly (*P<0.05, Wilcoxon rank-sum test) differentially regulated in the human and mouse angiosarcomas (VEGFA, VEGFB, KDR and MYC). Bars represent median, boxes show upper to lower quartiles, whiskers show the range excluding outliers. Grey, normal human and mouse endothelial cells; green, human and mouse angiosarcomas.

To understand the differences between the angiosarcomas that developed in the *Pdgfrb-Cre, Trp53^R172H/R172H^* and the *Cdh5-CreER^T2^, Trp53^fl/fl^* mice, we carried out gene expression analysis using the NanoString PanCancer Pathways panel. Tumour type-specific gene expression profiles were determined using unsupervised hierarchical clustering, with both tumour types also being significantly more different than normal VE-cadherin-derived endothelial cells (Fig. 6A). Gene ontology analysis of the genes that were significantly differentially expressed between the two different angiosarcoma subsets showed that pathways linked to p53 and angiogenesis, including FGF and VEGF signalling pathways, were over-represented (Fig. 6B). This indicates that the gain-of-function *Trp53^R172H^* mutant drives expression of a different set of genes to those seen in the *Cdh5-CreER^T2^, Trp53^fl/fl^* mice, to initiate angiosarcoma development.

To determine whether the changes seen in the differentially expressed genes in the mouse angiosarcomas reflect changes seen in human angiosarcomas, we carried out gene set enrichment analysis of the differentially expressed genes in the mouse angiosarcomas compared to normal endothelial cells and compared this with published human data from a set of human angiosarcomas and normal endothelial cells (GSE44115: Andersen et al., 2013). This showed a significant enrichment of genes associated with human angiosarcomas in the mouse tumours compared to those expressed in the normal endothelial cells (Fig. 6C), indicating that the mouse angiosarcomas represent a sub-population of human angiosarcomas. Interestingly, analysis of genes that are associated with endothelial cell function showed that a number were significantly increased in angiosarcomas in both the human and mouse datasets (VEGFC, EPHA2), whereas others were differentially regulated in the human and mouse angiosarcomas (VEGFA, VEGFB, KDR, MYC) (Fig. 6D).

### Generation of cell lines and transplantation model

**Fig. 7.**
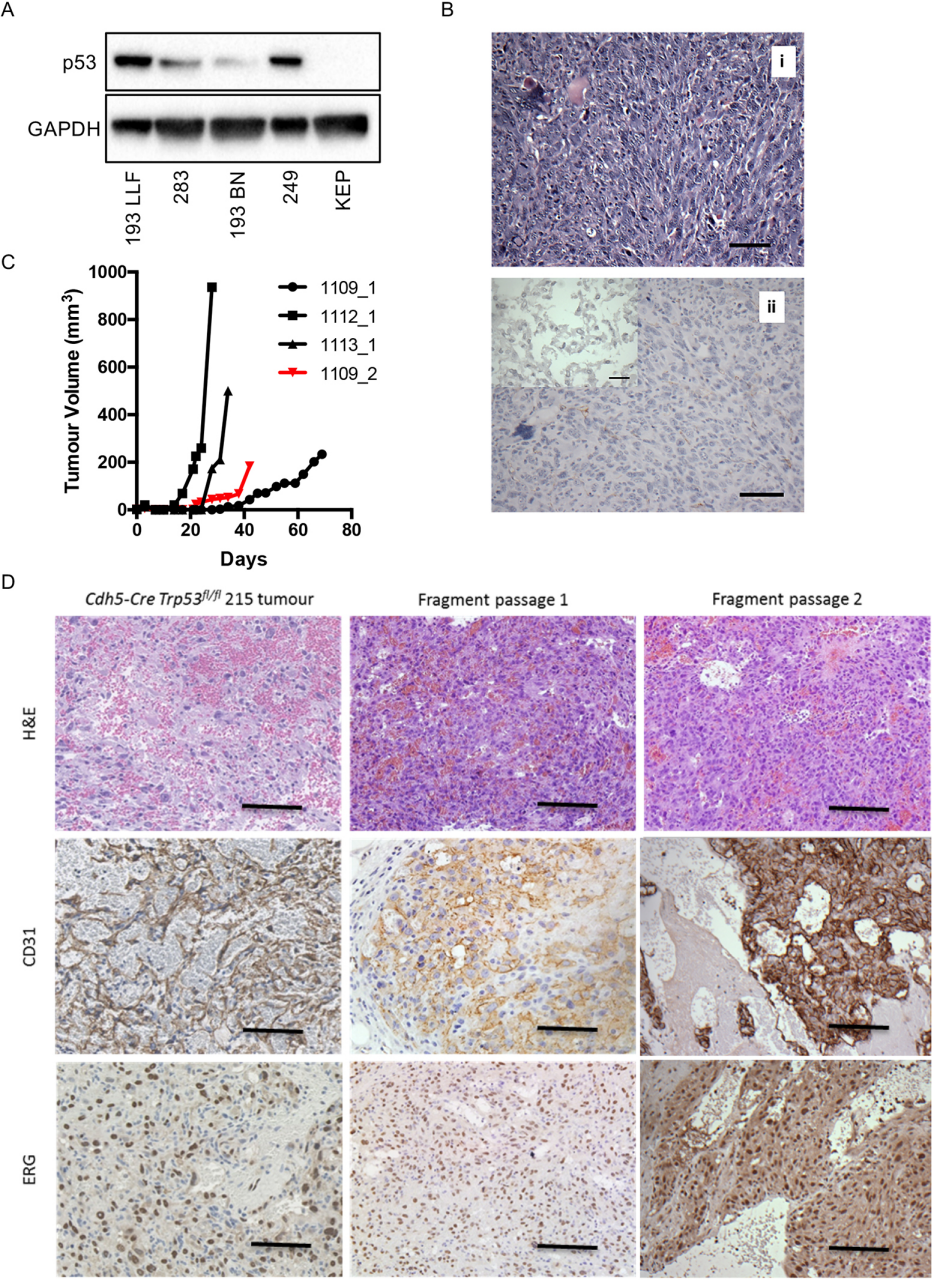
**Characterization of cell lines and fragment-derived tumours from mouse angiosarcomas.** (A) Western blot analysis of p53 expression in four *Pdgfrb-Cre, Trp53^R172H/R172H^* angiosarcoma-derived cell lines. Cell line nomenclature represents the mouse number from which the cell line was derived. KEP is a mouse mammary tumour cell line that is deficient in p53 owing to homozygous expression of a floxed p53 allele (Derksen et al., 2006). GAPDH was used as a loading control. (B) The morphology of tumours developing following implantation of angiosarcoma-derived cell lines was that of undifferentiated pleomorphic sarcomas. H&E staining (i) and immunohistochemical analysis using antibody to CD31 (ii). The tumour cells were negative for CD31, as was the implanted angiosarcoma-derived cell line (inset). (C) Growth of tumour fragments taken from three different *Cdh5-CreER^T2^, Trp53^fl/fl^* mice (1109, 1112, 1113) following subcutaneous transplantation into wild-type mice. 1109_2 is the secondary transplantation of fragments derived from tumour 1109_1. (D) H&E staining (top) and immunohistochemistry (CD31, middle; ERG, bottom) of *Cdh5-CreER^T2^, Trp53^fl/fl^* fragment-derived tumours. Left-hand panels show the spontaneous tumour, middle panels show the primary passage of tumour fragments and right-hand panels show the secondary passage of tumour fragments. Scale bars: 50 µm in B,D; 25 µm in inset, Bii.

We generated cell lines from four angiosarcomas that developed in the *Pdgfrb-Cre, Trp53^R172H/R172H^* mice. As with the spontaneous tumours, the cell lines all expressed p53 (Fig. 7A) and genotyping showed that each cell line was homozygous for the R172H allele. Upon re-implantation of the cell lines into the flanks of mice, two of the lines from the *Pdgfrb-Cre, Trp53^R172H/R172H^* mice developed tumours, with morphological features of undifferentiated pleomorphic sarcomas (Fig. 7Bi). Neither the generated cell lines nor the tumours retained expression of CD31 (Fig. 7Bii). We also generated cell lines from four angiosarcomas that developed in the *Cdh5-CreER^T2^, Trp53^fl/fl^* mice. Genotyping confirmed that each cell line was homozygous for the floxed *Trp53* allele. However, none of the cell lines formed tumours when injected into the flanks of recipient mice. In an attempt to overcome the loss of endothelial markers upon culture of the angiosarcomas we implanted tumour fragments from three spontaneous angiosarcomas that developed in the *Cdh5-CreER^T2^, Trp53^fl/fl^* mice. All formed tumours and histological examination confirmed that these were angiosarcomas expressing both CD31 and ERG (Fig. 7C,D). Furthermore, secondary implantation of frozen tumour fragments resulted in successful outgrowth of angiosarcomas in recipient wild-type mice (Fig. 7C,D).

## DISCUSSION

We have generated two mouse models of angiosarcoma driven by deregulation of p53. We used the *Pdgfrb-Cre* mouse that is known to target both pericytes and endothelial cells during development. This resulted in 75% of *Pdgfrb-Cre, Trp53^R172H/R172H^* mice developing angiosarcomas, which is higher than the 62% reported when *Tie2-Cre* mice were crossed to *Trp53* floxed mice (Farhang Ghahremani et al., 2014). In this model p53 is deleted in both endothelium and the haematopoietic lineages. Interestingly, we saw no angiosarcomas in the *Pdgfrb-Cre, p53^fl/fl^* mice suggesting that the *Trp53^R172H^* mutant is exerting a gain-of-function activity that is required for angiosarcoma development when *Pdgfrb* cells are targeted. The development of angiosarcomas in a small percentage of mice in a model of Li-Fraumeni syndrome that expresses *Trp53^R172H^* supports the specific involvement of mutant p53 in the development of angiosarcoma (Lang et al., 2004; Olive et al., 2004). Analysis of *Pdgfrb-Cre* mice has shown that recombination occurs in a number of cell types during development, including endothelial and mural cells (Stanczuk et al., 2015; Ulvmar et al., 2016), so it is not possible to define the cell of origin in the angiosarcomas that developed in the *Pdgfrb-Cre, Trp53^R172H/R172H^* mice.

The cell of origin of sarcomas remains unclear, although mounting evidence suggests that they are derived from mesenchymal cells (Yang et al., 2014). NG2 is a cell-surface proteoglycan expressed by pericytes, which are mesenchymal cells that surround blood vessels (Sá da Bandeira et al., 2017). A recent study has shown that targeting p53 loss in adult mice using NG2-driven Cre recombination leads to the formation of bone and soft tissue sarcomas, which supports the mesenchymal origin of these tumours (Sato et al., 2016). In this study only one angiosarcoma developed, indicating that they can arise from mesenchymal precursors but that the efficiency is much lower than that of other sarcoma types such as osteosarcomas and undifferentiated pleomorphic sarcomas, which were the most frequent tumour types seen. Interestingly, we found that loss of p53 in the *Pdgfrb-Cre* mice gave rise to undifferentiated sarcomas that expressed PDGFR-β, albeit with a reduced efficiency and increased latency compared to that observed by Sato and colleagues (Sato et al., 2016). This disparity may reflect differences in the efficiency of recombination in the *Pdgfrb-Cre* and *Ng2-Cre* mice or may be due to distinct pericyte subpopulations that are marked by PDGFR-β and NG2 during development (Birbrair et al., 2013). In the future, use of a conditional *Pdgfrb-iCreER^T2^* mouse (Claxton et al., 2008) that allows specific deletion of *Trp53* in the adult, in which *Pdgfrb* expression is restricted to pericytes, will allow further evaluation of the role of distinct mesenchymal cell lineages to the development of different sarcoma subtypes.

To address whether direct targeting of adult endothelial cells would result in the development of angiosarcomas with higher efficiency we used the *Cdh5-CreER^T2^* mouse. Surprisingly, all *Cdh5-CreER^T2^, Trp53^R172H/R172H^* mice developed lymphomas. Studies in *Cdh5-CreER^T2^* mice have reported recombination of a small (0.3%) subpopulation of bone marrow cells that are haematopoietic in nature (Monvoisin et al., 2006). Combined with the propensity of mutant p53 to drive lymphomagenesis, this appears to be sufficient to drive development of lymphomas in the *Cdh5-CreER^T2^, Trp53^R172H/R172H^* mice. However, all of the *Cdh5-CreER^T2^, Trp53^fl/fl^* mice developed angiosarcomas, with no lymphomas detected. The enrichment of genes associated with p53 in the differentially expressed genes between the tumours that develop in the two models indicates that, as expected, the gain-of-function *Trp53^R172H^* mutant drives expression of a different set of genes to those seen in the *Cdh5-CreER^T2^, Trp53^fl/fl^* mice to initiate angiosarcoma development. Other mouse models targeting specific endothelial cell populations have also been reported. mTORC1 activation in endothelial cells following conditional deletion of its upstream inhibitor *Tsc1* resulted in 100% of mice developing hepatic haemangiomas, and ∼80% developing cutaneous lymphangiosarcomas (Sun et al., 2015). This is in contrast to the aggressive angiosarcomas that developed upon direct targeting of *Trp53* in endothelial cell lineages in the *Cdh5-CreER^T2^, Trp53^fl/fl^* mice. mTORC1 pathway activation has also been reported in human angiosarcomas (Sun et al., 2015; Chadwick et al., 2018) and has been linked to Myc-mediated transcriptional regulation of VEGFA (Sun et al., 2015); increased expression of both *Myc* and *Vegfa* in the angiosarcomas from the *Cdh5-CreER^T2^, Trp53^fl/fl^* mice suggest that a similar autocrine stimulation loop may be present in these tumours, although the involvement of mTORC signalling in these tumours is not known. Combined loss of *Trp53*, *Pten* and *Ptpn12* also leads to the development of aggressive vascular lesions, which was associated with both mTORC and MEK pathway activation, suggesting that mTORC activation alone is not sufficient to drive aggressive angiosarcoma development (Chadwick et al., 2018). In this study, loss of *Trp53* alone did not result in the development of angiosarcomas. However, the tumours arose from more restricted recombination in a subset of endothelial cells. Taken together with the distinctive spectrum of tumours seen upon deletion of *Tsc1*, this suggests that targeting distinct populations of endothelial cells in mice in combination with differential pathway activation impacts on the type and site of vascular tumour development.

Increased Myc expression downstream of the forkhead boxO (FoxO) pathway has been linked to endothelial cell proliferation and angiosarcoma development (Riddell et al., 2018; Wilhelm et al., 2016). Interestingly, conditional triple knock-out of FOXO1/3/4 in mice results in development of thymic lymphomas and vascular lesions, predominantly haemangiomas, in a number of tissues, with only 9% progressing to angiosarcomas, although there is no reported role for Myc in this model (Paik et al., 2007). This is in contrast to the *Cdh5-CreER^T2^, Trp53^fl/fl^* mice, in which angiosarcomas arise predominantly in soft tissues, as the vascular lesions that develop in the FOXO triple knockout mice are predominantly benign and arise in relation to the uterus and a number of other tissues (Paik et al., 2007). FOXOs and p53 share many common target genes and may act in a cooperative manner in order to regulate gene transcription (Fu and Tindall, 2008; Renault et al., 2011). It will be interesting to establish whether the deregulation of such pathways is required for driving the transition from benign to malignant disease in the mouse models.

Angiosarcomas in humans may be divided into several clinical groups. The majority of cases (∼50%) are cutaneous, breast parenchymal angiosarcomas account for ∼14%, soft tissue 11%, heart 7% and bone 4%, with a range of other sites accounting for the remainder (Lahat et al., 2010). Little is known of the factors that predispose to angiosarcoma development in the clinical setting, and it may be that angiosarcomas constitute a range of interrelated clinical diseases with common endothelial features but different phenotypes and aetiologies. Comparison of gene expression profiles between the two mouse models and human angiosarcomas showed significant enrichment of angiosarcoma-associated genes in the two models, suggesting that the mouse models do represent some of the phenotypes present in the human disease. Use of these and other mouse models will help to unravel some of the targetable pathways in angiosarcoma.

Although genetically engineered models are used in preclinical studies, the long latency of tumour formation in the *Cdh5-CreER^T2^, Trp53^fl/fl^* mice (median lifespan 325 days) and the development of multiple tumours would make therapeutic efficacy studies costly and challenging. Knockout of p53 in alymphocytic *Rag2^−/−^;Il2rg^−/−^* mice leads to a high frequency of angiosarcomas (over 65%), with only sporadic formation of lymphomas. With a mean latency of 18 weeks, this provides an alternative model that would be more amenable to therapeutic studies (Landuzzi et al., 2014). However, the lack of immune cell populations in these mice restricts their use for assessing immune modulators, which are showing promise in sarcoma. A recent report has shown that use of a lentiviral vector-based system to introduce oncogenic *Hras^G12V^* in combination with loss of *Cdkn2a* via intravenous injection into immune competent mice resulted in the formation of angiosarcomas. These develop very rapidly and in multiple sites, which is most likely influenced by the intravenous route of injection (Yang et al., 2014). This provides a useful model for preclinical studies, but the rapid development of multiple tumours will make these studies challenging.

To overcome these issues, we generated cell lines from angiosarcomas that developed in the *Pdgfrb-Cre, Trp53^R172H/R172H^* and *Cdh5-CreER^T2^, Trp53^fl/fl^* mice, in an attempt to generate syngeneic mouse models of angiosarcoma. However, as has been reported previously (Farhang Ghahremani et al., 2014), endothelial markers were rapidly lost when the tumours were established in culture, even when grown with endothelial cell-specific supplements. In contrast, direct implantation of tumour fragments allowed us to establish angiosarcomas which could be frozen and passaged serially through wild-type recipient mice. This had the benefit of reducing the latency of tumour formation and restricting the number of tumours per mouse. Furthermore, the subcutaneous localization of the tumours allowed easy monitoring of tumour growth. We have previously used this transplantation approach to model HER2 breast cancer and have demonstrated its utility in determining drug efficacy and establishing models of drug resistance (Creedon et al., 2016).

Taken together, our data indicate that deleting p53 in endothelial cells in the adult mouse is the most effective way to generate angiosarcomas. This resulted in 100% penetrance with no formation of lymphomas. The development of lymphomas in the *Cdh5-CreER^T2^, Trp53^R172H/R172H^* mice supports a strong selection for angiosarcoma formation following loss of p53 in contrast to expression of the gain-of-function mutant p53. This is supported by angiosarcoma formation in mice in which loss of *Trp53* is combined with loss of *Ptpn12* and *Pten* (Sá da Bandeira et al., 2017). The further establishment of a transplantation model of angiosarcoma provides a novel approach for testing potential new therapeutics in this disease setting.

## MATERIALS AND METHODS

### Animals

Mice expressing *Cre* under the control of the *Pdgfrb* promoter (*Pdgfrb-Cre*) (Foo et al., 2006) or in the inducible control of the *Cdh5* promotor (*Cdh5-CreER^T2^*) (Wang et al., 2010) were crossed to mice expressing either a mutant p53*^R172H^* (Olive et al., 2004) or floxed p53 allele (Jonkers et al., 2001) to give experimental cohorts on a mixed background, segregating for C57BL/6J and S129 genomes. The mutant p53 allele is preceded by a STOP cassette, flanked by *loxP* sites, such that upon activation of Cre recombinase the STOP cassette is excised and the mutant p53*^R172H^* is expressed, whereas in the floxed p53 mice activation of Cre deletes exons 2-10, resulting in a loss of p53. *Pdgfrb-Cre* and *Cdh5-CreER^T2^* mice were also crossed with Ai14 (Rosa-CAG-LSL-tdTomato-WPRE) mice (Madisen et al., 2010), obtained from the Jackson Laboratory, to allow endogenous reporting in *Pdgfrb*- and *Cdh5*-expressing cells. Genotyping was carried out by Transnetyx. *Cdh5-CreER^T2^* mice were treated with tamoxifen (Sigma-Aldrich) (100 mg/kg body weight, intraperitoneal injection) for 5 days at 6 weeks of age. Mice were monitored twice weekly and sacrificed when cutaneous tumours had reached a maximum size of 1.5 cm or the mouse became sickly, as defined by UK Home Office guidelines. Following sacrifice, macroscopically identified tumours and major organs were removed and fixed in 10% neutral buffered formalin. In some instances, fresh samples were taken for generation of cell lines or tumour fragments taken for re-implantation. Animal studies and procedures were approved by the University of Edinburgh Ethical review committee (Application #PL01-16) and conducted in accordance with United Kingdom Home Office regulations.

### Histology and immunohistochemistry

Formalin-fixed tissues were routinely processed into paraffin wax blocks and sections cut for Haemotoxylin and Eosin (H&E) staining and immunohistochemistry. Immunohistochemistry was carried out as described previously (Creedon et al., 2016). Primary antibodies used were CD31 at 1:800 (Abcam, ab28364), ERG (Dako, IR659, ready to use, 200 μl per slide), p53 at 1:2000 (Leica BioSystems, NCL-L-p53-CM5p), PDGFR-β at 1:100 (CST, 3169S), and VE-cadherin at 1:4000 (Abcam, ab33168).

### Immunofluorescence

Shaved dorsal skin was mounted on 3 mm blotting paper and placed in 4% methanol-free formaldehyde (Thermo Fisher Scientific, 28906) at 4°C for 1 hour. The skin was then washed with PBS and transferred to 18% sucrose overnight for cryoprotection before embedding in OCT embedding matrix (Cellpath, KMA-0100-00A) and frozen on dry ice. Then 7 µm cryotome sections were dried at room temperature in the dark for 30 min and washed in PBS containing 0.05% Tween 20 (Sigma-Aldrich, P2287). To image endogenous tdTomato fluorescence, sections were incubated with 1 µM DAPI for 10 min, washed twice with PBS and mounted with Prolong Gold (Thermo Fisher Scientific, P36930). For indirect immunofluorescence staining, sections were incubated with blocking buffer [PBS containing 5% goat serum (Vector, S-1000) and 0.3% Triton X-100 (Sigma-Aldrich, T8787)] for 30 min, then incubated with primary antibodies PDGFR-β (1:25, Abcam, Ab32570) or CD31 (1:50, BD Pharmingen, 550274) for 2 h. Sections were then washed twice and incubated for 30 min with Alexa fluor 488 conjugated secondary antibodies [Molecular Probes; goat anti-rabbit 488 (1:1000, A11034) or goat anti-rat 488 (1:1000, A11006)], washed twice, incubated with DAPI and mounted as described above. Images were obtained using a Zeiss LSM780 confocal microscope.

### Cell culture

Tumours from *Pdgfrb-Cre, p53^R172H/R172H^* mice were freshly processed by rinsing in PBS and then mincing to ∼1 mm^3^ pieces using two scalpels. After transferring to a 15 ml Falcon tube containing 10 ml digestion media [maintenance medium omitting foetal bovine serum (FBS) and including 300 U/ml collagenase I (Worthington) and 100 U/ml hyaluronidase (Fisher)], cells were subjected to shaking incubation at 37°C for 18 h. After pelleting at 1300 rpm (340 ***g***) for 5 min, cells were resuspended in 2 ml maintenance media [DMEM/Ham's F12 (Sigma-Aldrich), 10% FBS (Gibco), amphotericin (Gibco) and penicillin-streptomycin (Sigma-Aldrich)] before being transferred to a six-well plate. Cells were left undisturbed for 5-7 days in a humidified incubator at 37°C with 5% CO_2_ before refreshing media and moving on to flasks when confluent. Tumours from *Cdh5-CreER^T2^, Trp53^fl/fl^* mice were manually minced using scalpels and left undisturbed for 5-7 days before transferring to flasks when confluent. Cells were maintained in Ham's F-12K Medium (Gibco), 10% FBS (Gibco), amphotericin B (Gibco), penicillin-streptomycin (Sigma-Aldrich), 0.1 mg/ml heparin (Sigma-Aldrich) plus 20 µg/ml endothelial cell growth supplement (Sigma-Aldrich, E2759) and checked routinely for mycoplasma infection.

### Western analysis

SDS-denatured protein samples made from RIPA lysates were run on 4%-15% precast polyacrylamide gels (Bio-Rad) in Tris/Glycine/SDS running buffer (Bio-Rad) and transferred onto nitrocellulose using the Trans-Blot^®^ Turbo System (Bio-Rad). After blocking the membrane in 5% bovine serum albumin in PBS, anti-p53 antibody (VectorLabs, VP-P955) was added at 1:50 overnight at 4°C on a shaking platform. The membrane was washed in TBS-T thrice before being incubated with an HRP-conjugated anti-rabbit secondary antibody (Cell Signaling Technology, #7074, 1:1000). Washes were repeated and then the membrane visualized on a ChemiDoc (Bio-Rad) using ECL western blotting substrate (Pierce).

### Gene expression profiling

RNA prepared from angiosarcomas that developed in the *Pdgfrb-Cre, p53^R172H/R172H^* and *Cdh5-CreER^T2^, Trp53^fl/fl^* mice was analyzed using the NanoString PanCancer Pathways panel (represents 750 cancer associated genes) on the NanoString nCounter DX platform as per the manufacturer's instructions. For comparison, VE-cadherin-positive liver endothelial cells were isolated from Ai14;*Cdh5-CreER^T2^* mice 3 weeks after tamoxifen treatment (Lynch et al., 2018). A VE-cadherin (tdTomato)-positive population of endothelial cells was collected using a BD FACSAria II. Following standard nCounter normalisation, differentially expressed genes were identified using Student's *t*-tests (*P*<0.05) between the angiosarcomas derived from the *Pdgfrb-Cre, p53^R172H/R172H^* and *Cdh5-CreER^T2^, Trp53^fl/fl^* mice and relative to the normal endothelial cells. Hierarchical cluster analysis was performed with the Cluster and Treeview programs (Eisen et al., 1998). Gene set enrichment analysis (Subramanian et al., 2005) was performed using the Phenotest R package. Gene ontology analysis was performed using the PANTHER classification system (Paik et al., 2007).

### Sub-cutaneous tumour growth

Cell lines and tumour fragments derived from the mouse angiosarcomas were injected into both flanks of 6- to 8-week-old female CD-1 nude mice (Charles River) and tumour growth measured twice weekly using calipers. Tumour volumes were calculated in Excel using the formula v=4/3π*r*^3^. Animals were sacrificed when tumours reached the maximum size allowed, and collected and fixed in 10% neutral buffered formalin.

## Supplementary Material

Supplementary information
