# Supplementary material for: Development of mouse models of angiosarcoma driven by p53
Source: Dis Model Mech. 2019 Jul 9;12(7):dmm038612. doi: 10.1242/dmm.038612 (PMC6679377; doi:10.1242/dmm.038612)
Supplement: Supplementary information [file dmm-12-038612-s1.pdf]

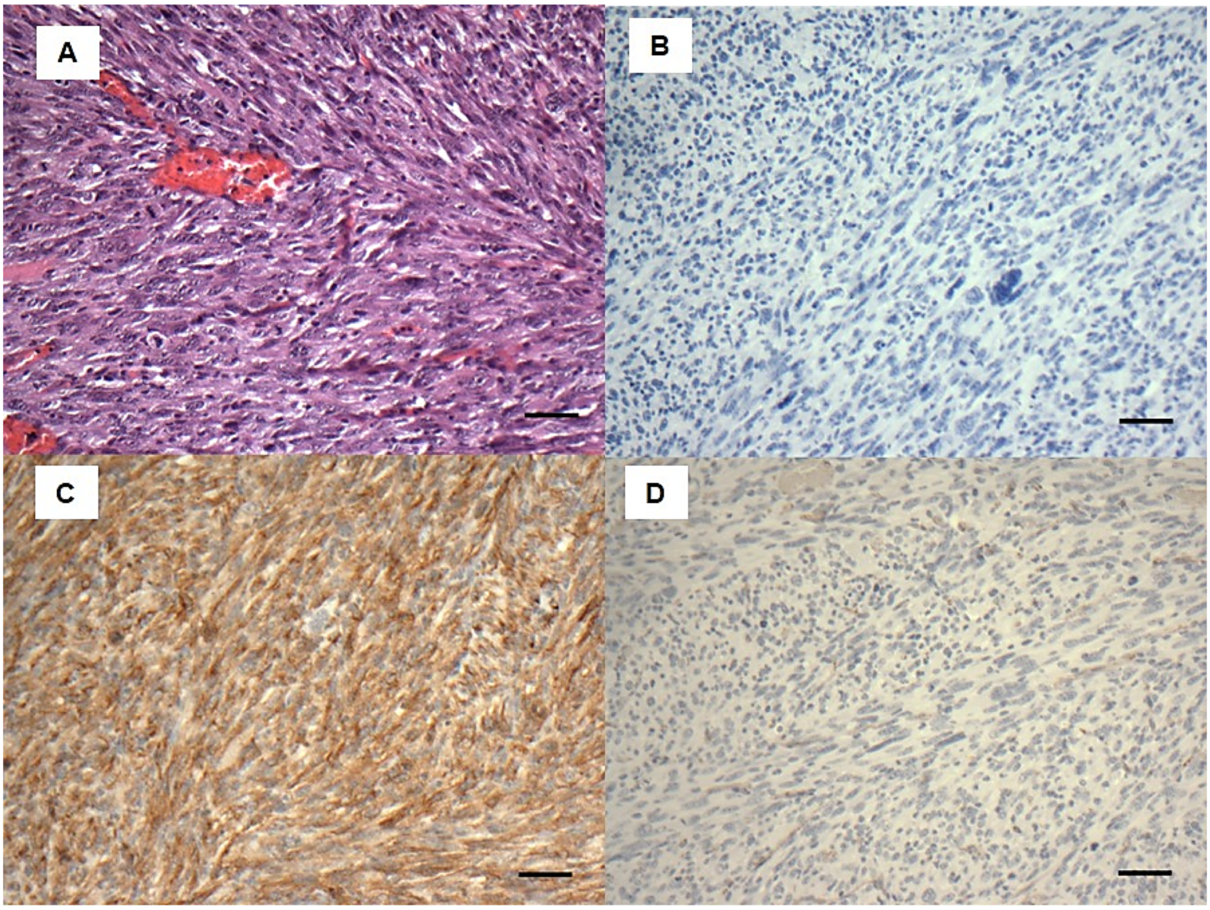

Figure S1. Immunohistochemistry of mouse undifferentiated sarcomas. (A) H&E staining showed tumours comprising spindle and pleomorphic cells without evidence of specific lineage differentiation histologically. Cells were (B) negative for p53, (C) positive for PDGRFβ and (D) negative for CD31. Scale bars = 50 μm.

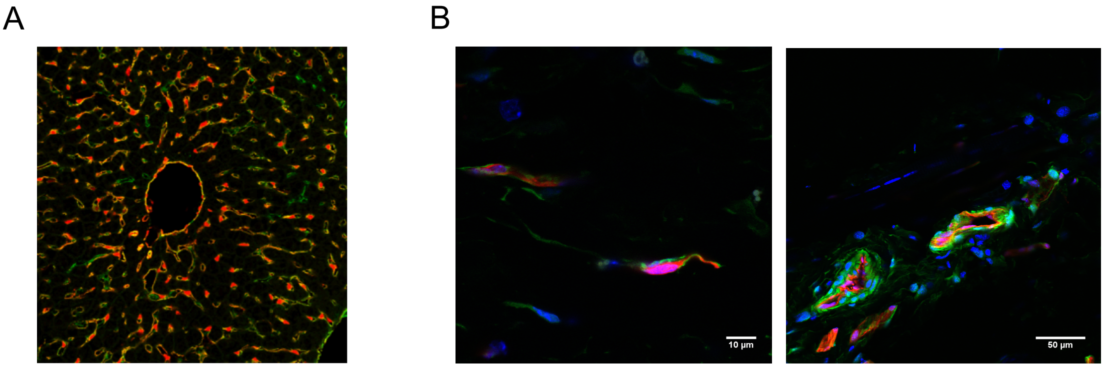

Figure S2. Confocal micrographs of fixed frozen liver (A) and dorsal skin (B) from *Cdh5-CreER*^{T2} mouse crossed with a tdTomato floxed reporter (*Ai14; Cdh5-CreER*^{T2}) showing cytosolic tdTomato fluorescence (red) and CD31 antibody cell surface staining (green). DAPI (blue) indicates nuclei.
